# Decellularized porcine vascular grafts: structural integrity and translational potential for allogeneic implantation

**DOI:** 10.3389/fbioe.2026.1823315

**Published:** 2026-05-26

**Authors:** Liqi Zhao, Henghua Fan, Bin Ding, Yuhui Ji, Feng Wang, Weihai Jiang, Zhenyong Wang, Wenyuan Wu, Jing Shi, Zhenguo Yan

**Affiliations:** 1 Department of Orthopedic Surgery, The Fourth People’s Hospital of Shenzhen (Shenzhen Samii Medical Center), Shenzhen, China; 2 Department of Orthopedic Surgery, Shenzhen Bao’an Shiyan People’s Hospital, Shenzhen, China

**Keywords:** allogeneic implantation, decellularized vascular grafts, extracellular matrix, porcine carotid artery, recellularization, vascular tissue engineering

## Abstract

Decellularized vascular grafts (DVGs) are a promising alternative to synthetic prostheses for applications requiring long-term patency and biological integration. Here, we assessed porcine decellularized carotid arteries as vascular grafts using a detergent-enzymatic protocol. We removed cellular components while preserving native extracellular matrix architecture. Histology and immunohistochemistry confirmed effective removal of endothelial and smooth muscle cells, with intact collagen and elastin. Residual DNA content (32.6 ± 5.8 ng/mg) fell below the 50 ng/mg immunogenicity threshold. Mechanical testing showed tensile properties and burst pressure comparable to native vessels. *In vitro* cytotoxicity assays demonstrated favorable cytocompatibility. Hemocompatibility evaluation revealed a hemolysis ratio below 5%, coagulation parameters within normal ranges, and limited platelet adhesion with non-activated morphology. *In vivo* allogeneic implantation over 3 months showed graft patency without thrombosis or structural failure. Explanted grafts exhibited partial endothelial coverage, cellular infiltration, and smooth muscle cell migration, indicating active host-mediated remodeling. These findings indicate that porcine decellularized carotid arteries preserve essential architecture, biomechanics, bioactivity, and hemocompatibility required for vascular reconstruction, highlighting their translational potential as biologically functional and scalable vascular grafts.

## 1 Introduction

Cardiovascular disease has become the leading cause of mortality worldwide, driven by rising living standards and an aging population. Vascular bypass grafting remains the optimal treatment for long-term revascularization, with an urgent clinical need for small-caliber (inner diameter <6 mm) grafts, particularly in coronary and peripheral artery diseases (Campbell et al., 2012). Traditional synthetic grafts fail to maintain long-term patency due to issues including thrombosis, infection and inflammation, luminal stenosis, and immunogenicity-related rejection risks. This is particularly evident in small-diameter applications where failure is commonplace (Pashneh-Tala et al., 2016). Non-degradable synthetic vascular grafts pose persistent risks to patients regarding patency rates, mechanical compliance, infection susceptibility, and durability (Hussein et al., 2016).Autologous veins, such as the great saphenous vein, remain the gold standard for arterial repair, offering good patency and low infection rates. Nevertheless, their availability is limited by patient comorbidities or prior harvesting, and their use carries risks of intimal hyperplasia and thrombosis (Velez et al., 2025). Decellularized vascular grafts (DVGs) have emerged as a biologically derived alternative that preserves native extracellular matrix (ECM) architecture while reducing immunogenicity. Porcine arteries are particularly attractive due to their anatomical and mechanical similarity to human vessels, facilitating translational development (Ma et al., 2012). Here, we assessed porcine decellularized carotid arteries through histological, mechanical, hemocompatibility, and *in vivo* analyses, providing preliminary observations and identifying remaining challenges for further development.

## 2 State of the art of DVGs

### 2.1 Clinical limitations of existing vascular grafts

Autologous vessels remain the gold standard for vascular reconstruction; however, their availability is limited by patient comorbidities and prior harvesting. Synthetic vascular grafts, such as expanded polytetrafluoroethylene and polyethylene terephthalate, demonstrate acceptable performance in large-diameter applications but are associated with thrombosis, intimal hyperplasia, and poor long-term patency in small-diameter settings (Wang et al., 2023). Emerging technologies including 3D bioprinting (Kim et al., 2020), electrospinning (Wu Y. et al., 2025), and cell-seeded scaffolds demonstrate potential in creating personalised grafts. However, scalability and long-term stability remain to be observed. Recent studies have even reported plant-derived scaffolds, involving the decellularisation of Viburnum opulus leaves to construct plant-derived extracellular matrix scaffolds, offering a scalable and biocompatible alternative for synthetic grafts and autologous vessels (Imeidopf et al., 2025). Nevertheless, these non-biological synthetic transplant materials have not achieved significant progress in biocompatibility and struggle to maintain long-term stable patency. These limitations have driven the development of biologically derived grafts capable of recapitulating native vascular structure and function.

### 2.2 Progress in decellularization strategies for vascular tissues

Decellularization has emerged as a promising approach to generate extracellular matrix (ECM)-based vascular scaffolds by removing cellular components while preserving tissue-specific architecture (Heath, 2019; Naegeli et al., 2022). Current strategies generally involve combinations of physical (Fitriatul et al., 2017), chemical, and enzymatic treatments, each with distinct advantages and limitations. Detergent-based protocols effectively remove cellular remnants but may compromise ECM integrity if not carefully optimized (Funamoto et al., 2010). Consequently, increasing efforts have focused on balancing decellularization efficiency with preservation of biomechanical and biochemical cues essential for vascular regeneration.

### 2.3 Biological and mechanical advantages of DVGs

DVGs retain the hierarchical ECM organization of native vessels, including collagen and elastin networks that govern mechanical compliance and resilience. Importantly, the preserved ECM provides bioactive signals that facilitate endothelial cell adhesion, migration, and functional maturation. Previous studies have demonstrated that ECM-based grafts can promote *in situ* endothelialization and smooth muscle cell remodeling, thereby reducing thrombogenicity and improving long-term patency compared with synthetic alternatives.

### 2.4 Remaining challenges and translational barriers

Despite encouraging preclinical and early clinical outcomes, several challenges hinder the widespread translation of DVGs. These include variability in decellularization outcomes, incomplete recellularization, immune modulation following implantation, and uncertainty regarding long-term mechanical durability. Moreover, systematic evaluations combining histological validation, mechanical characterization, cytocompatibility, and *in vivo* implantation remain limited, particularly for large-animal–derived grafts intended for clinical translation (Dahan et al., 2017). In this context, porcine arteries represent an attractive source owing to their anatomical similarity to human vessels and established translational relevance.

## 3 Materials and methods

### 3.1 Source of porcine arteries

Porcine carotid arteries were harvested from healthy adult Large White pigs under sterile conditions. Immediately after excision, the vascular lumen was flushed with sterile 0.9% sodium chloride solution containing 50 IU/mL heparin to remove residual blood. Surrounding connective tissues were carefully removed. The cleaned arteries were then immersed in sterile phosphate-buffered saline (PBS) and stored at −80 °C for 1 week prior to decellularization. This freeze–thaw process served as a physical pretreatment to induce ice crystal formation and disrupt cellular membranes, thereby facilitating subsequent decellularization. All animal procedures were approved by the Animal Ethics Committee of Kerbio Medical Testing Co., Ltd. (Approval No. IACU24-0005) and were conducted in accordance with institutional guidelines and the National Institutes of Health (NIH) Guide for the Care and Use of Laboratory Animals.

### 3.2 Decellularization protocol

Decellularized porcine carotid arteries were prepared using a multistep detergent–enzymatic protocol designed to efficiently remove cellular components while preserving extracellular matrix architecture. This approach was optimized to maintain mechanical integrity and biological cues required for subsequent *in vivo* implantation and vascular remodeling. This protocol was optimized through extensive preliminary testing to balance efficient cellular removal with ECM integrity, aiming to support potential allogeneic and xenogeneic vascular transplantation. Briefly, frozen porcine carotid arteries were thawed at room temperature and subjected to the following sequential treatments:

Step (a): The arteries were immersed in deionized water and continuously agitated for 12 h to create a hypotonic environment, inducing osmotic lysis of cells and release of intracellular contents.
Step (b): After rinsing three times with PBS (15 min each), the arteries were treated with deionized water under agitation for an additional 4 h, followed by incubation in 1% (v/v) Triton X-100 solution prepared in Tris–HCl buffer (pH 8.0) for 24 h to solubilize cell membranes.
Step (c): The tissues were rinsed three times with PBS to remove residual detergent and subsequently treated with 0.125% trypsin solution under continuous agitation for 1 h to further degrade cellular remnants.
Step (d): After PBS washing, the arteries were incubated in 1% sodium dodecyl sulfate (SDS) solution for 24 h. This ionic detergent was used to disrupt nuclear membranes, cytoskeletal structures, and non-matrix proteins tightly associated with the ECM.
Step (e): The tissues were agitated in deionized water for 4 h to thoroughly remove residual SDS, followed by treatment with DNase solution (20–30 U/mL) for 1 h to degrade residual nucleic acids. The DNase solution was freshly prepared and used within 2 h to ensure enzymatic activity.
Step (f): Finally, the arteries were rinsed three times with PBS (15 min each) to remove enzymatic residues and degradation products, and further washed twice in Hanks’ balanced salt solution, with fresh solution replaced every 4 h.

All decellularization steps were performed on an orbital shaker at a controlled agitation speed of 80–120 rpm. During each incubation step, the respective solutions were replaced every 8 h to prevent saturation and maintain decellularization efficiency. To prevent ECM degradation by endogenous proteases and microbial contamination, protease inhibitors and antibiotics were added to all aqueous solutions. Specifically, phenylmethylsulfonyl fluoride (PMSF, 1–5 μM) was used as a protease inhibitor, and a combination of cefazolin sodium (1 mg/mL) and streptomycin (100 μg/mL) was added as antibiotic agents. The pH of all buffers (PBS, Tris-HCl, and Hanks’ balanced salt solution) was verified and adjusted to the specified values (pH 7.4 for PBS, pH 8.0 for Tris-HCl) using a calibrated pH meter before each use.

### 3.3 Residual DNA quantification

Residual double-stranded DNA (dsDNA) content was quantified in both native porcine carotid arteries (fresh artery, n = 5) and DVGs (n = 5) to assess decellularization efficiency and potential immunogenicity. Tissue samples were lyophilized using a freeze-dryer (Alpha 1–2 LDplus, Martin Christ, Germany) and weighed to obtain dry tissue weight (10–15 mg per sample). Each sample was digested overnight at 56 °C in a lysis buffer containing proteinase K (20 mg/mL, Thermo Fisher Scientific, United States). Total DNA was extracted using a DNeasy Blood & Tissue Kit (Qiagen, Hilden, Germany) according to the manufacturer’s protocol. The concentration of dsDNA was measured fluorometrically using the Quant-iT™ PicoGreen™ dsDNA Assay Kit (Invitrogen, United States) with a microplate reader (SpectraMax M5, Molecular Devices, United States) at excitation/emission wavelengths of 480/520 nm. A standard curve was generated using lambda DNA standards supplied with the kit. DNA content was calculated as nanograms of DNA per milligram of dry tissue weight (ng/mg dry tissue). All measurements were performed in triplicate.

### 3.4 Hemocompatibility evaluation

Hemocompatibility of the DVGs was assessed through platelet adhesion observation, hemolysis testing, and coagulation assays, following the guidelines of ISO 10993-4.

Platelet adhesion assay: Fresh rabbit blood was collected using sodium citrate as an anticoagulant (9:1 blood-to-citrate ratio). Platelet-rich plasma (PRP) was obtained by centrifuging the blood at 1,500 rpm for 15 min. DVG samples (0.5 cm × 0.5 cm, rehydrated in PBS) were incubated with 500 μL of PRP at 37 °C for 1 h. After gentle washing with PBS to remove non-adherent platelets, the samples were fixed with 2.5% glutaraldehyde, dehydrated through a graded ethanol series, and sputter-coated with gold. Platelet morphology and adhesion were examined using scanning electron microscopy (SEM, Hitachi S-4800, Japan).

Hemolysis assay: Fresh rabbit blood anticoagulated with sodium citrate (9:1 volume ratio) was used. Erythrocytes were isolated by adding physiological saline and centrifuging at 1,500 rpm for 15 min. After discarding the supernatant, the erythrocytes were washed with physiological saline and centrifuged at 1,000 rpm for 5 min; this washing procedure was repeated 2–3 times until the supernatant became clear. The purified erythrocytes were diluted with sterile PBS to obtain a 5% (v/v) red blood cell suspension. For the negative control, 500 μL of the diluted blood suspension was mixed with 500 μL PBS. For the positive control, 500 μL of the diluted blood suspension was mixed with 500 μL deionized water. For the experimental group, a DVG sample (0.5 cm × 0.5 cm, rehydrated) was incubated with 500 μL of the diluted blood suspension. All groups were incubated at 37 °C for 2 h, followed by centrifugation at 1,000 rpm for 5 min. Subsequently, 100 μL of supernatant was transferred to a 96-well plate, and absorbance was measured at 540 nm using a microplate reader (SpectraMax M5, Molecular Devices, United States). The hemolysis ratio (H) was calculated as: H (%) = (A_sample − A_negative)/(A_positive − A_negative) × 100%.

Coagulation assays: Fresh rabbit blood was collected into sodium citrate tubes. Plasma was separated by centrifugation at 3,000 rpm for 10 min. Prothrombin time (PT) and activated partial thromboplastin time (aPTT) were measured using a fully automated coagulation analyzer (ACL Top 700, Instrumentation Laboratory, United States) according to the manufacturer’s instructions. Measurements were performed in triplicate for each sample (n = 5 per group). Normal reference ranges for rabbit plasma were used as controls.

### 3.5 *In vivo* allogeneic implantation

DVGs (length 2 cm, inner diameter 3–4 mm) were implanted as allogeneic vascular grafts in healthy adult Large White pigs (n = 5). Each animal received a single graft implanted into the carotid artery using end-to-end anastomosis under general anesthesia. No sham operation or synthetic graft control group was included in this study. After surgery, animals were monitored daily for signs of graft failure, infection, or thrombosis. Grafts were explanted at 3 months post-implantation for subsequent histological and morphological analyses. The sample size (n = 5) was chosen based on previous studies in the field and was sufficient for preliminary patency and remodeling assessment. No animals were lost to follow-up.

### 3.6 Statistical analysis

All quantitative data are presented as mean ± standard deviation (SD) from at least three independent experiments, unless otherwise stated. Sample sizes (n) for each analysis are specified in the respective figure legends and refer to the number of biological replicates (individual arteries) per group. Normality of data distribution was assessed using the Shapiro–Wilk test. For comparisons between two groups (e.g., native vs. decellularized), an unpaired two-tailed Student's t-test was applied when data followed a normal distribution; otherwise, the Mann–Whitney U test was used. For comparisons involving more than two groups, one-way analysis of variance (ANOVA) followed by Tukey’s *post hoc* test was performed. A p-value of less than 0.05 was considered statistically significant. All statistical analyses were conducted using GraphPad Prism 9.0 (GraphPad Software, San Diego, CA, United States). In all figures, error bars represent SD, and significance levels are indicated as *p < 0.05, **p < 0.01, and ***p < 0.001.

## 4 Results

### 4.1 Effective decellularization and preservation of vascular ECM

**FIGURE 1:**
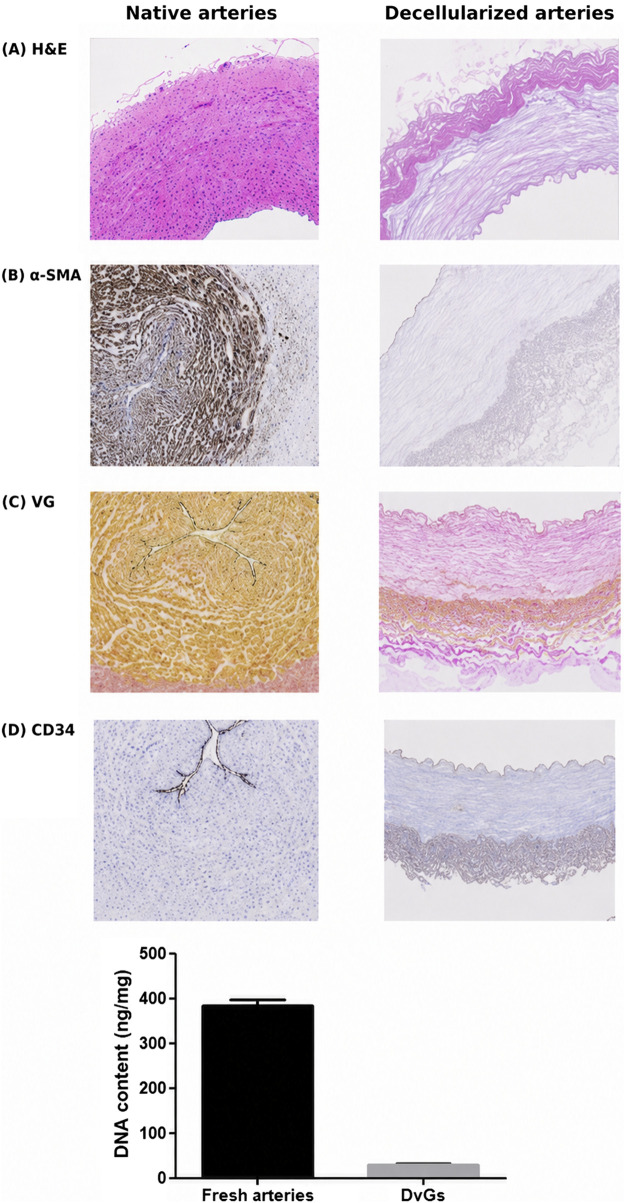
Histological and immunohistochemical assessment of decellularization in porcine carotid arteries. Representative images of Fresh artery and decellularized vascular grafts (DVGs): **(A)** H&E staining; **(B)** α-smooth muscle actin (α-SMA) immunostaining; **(C)** Van Gieson (VG) staining; **(D)** CD34 immunostaining. **(E)** Quantitative analysis of residual DNA content in native porcine carotid arteries (Fresh artery) and DVGs. Data are presented as mean ± SD (n = 5 per group). ***p < 0.001 (unpaired two-tailed Student's t-test).

Histological analyses were performed to evaluate the efficacy of the decellularization process and the preservation of extracellular matrix architecture. Hematoxylin and eosin staining demonstrated complete removal of cellular nuclei and cytoplasmic components in decellularized porcine carotid arteries compared with native tissues (Figure 1A). Immunohistochemical staining for α-smooth muscle actin confirmed the absence of smooth muscle cells following decellularization, whereas native arteries exhibited strong positive signals (Figure 1B). Van Gieson staining further revealed well-preserved collagen and elastin fibers within the vessel wall, indicating that the hierarchical ECM structure was largely maintained after treatment (Figure 1C). In addition, CD34 immunostaining showed effective removal of endothelial cells from the luminal surface (Figure 1D), confirming successful decellularization of all vascular layers.

Residual DNA content was quantified to evaluate the efficiency of decellularization and the potential immunogenicity of the scaffolds. As shown in Figure 1E, native porcine carotid arteries (Fresh artery) contained a high level of residual DNA (385.2 ± 42.1 ng/mg dry tissue, n = 5). In contrast, DVGs exhibited a significantly reduced DNA content of 32.6 ± 5.8 ng/mg dry tissue (n = 5, p < 0.001, unpaired two-tailed Student's t-test). This value falls below the widely accepted threshold of 50 ng/mg dry tissue, which is associated with substantially reduced host immune response upon implantation. These quantitative results, together with the absence of visible nuclei on histological examination (Figure 1E), confirm that the applied decellularization protocol efficiently removes cellular and nuclear materials. The low residual DNA content suggests that the prepared DVGs possess favorable characteristics for subsequent host integration and vascular remodeling with reduced immunogenicity.

### 4.2 Mechanical integrity of porcine arteries after decellularization

**FIGURE 2:**
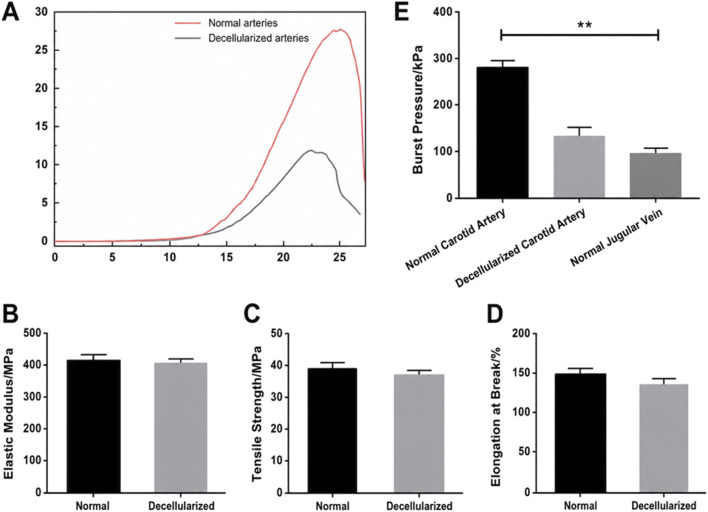
Mechanical characterization of native and decellularized porcine carotid arteries. **(A)** Representative stress–strain curves of native (black line) and decellularized (red line) arteries. **(B)** Elastic modulus (MPa). **(C)** Tensile strength (MPa). **(D)** Elongation at break (%). All data in **(B–D)** are presented as mean ± SD (n = 5 per group). No statistically significant differences were observed between native and decellularized carotid arteries for any of these parameters (unpaired two-tailed Student’s t-test, p > 0.05 for all comparisons). **(E)** Burst pressure comparison among native carotid artery, decellularized carotid artery, and native jugular vein (kPa). Data are presented as mean ± SD (n = 5 per group). One-way ANOVA followed by Tukey’s *post hoc* test revealed that the burst pressure of the native carotid artery was significantly higher than that of the native jugular vein (p < 0.001). No significant differences were observed between the native carotid artery and the decellularized carotid artery, nor between the decellularized carotid artery and the native jugular vein (p = 0.06). **p < 0.01.

The mechanical performance of decellularized porcine carotid arteries was evaluated using uniaxial tensile testing and two-dimensional burst pressure analysis. Stress–strain curves of decellularized grafts exhibited nonlinear behavior comparable to that of native arteries (Figure 2A), reflecting preserved elastic characteristics. Quantitative analysis demonstrated no significant differences in tensile strength, elastic modulus, or elongation at break between native and decellularized vessels (Figures 2B–D). Furthermore, burst pressure testing revealed that decellularized grafts withstood physiological pressure levels without structural failure (Figure 2E). These results indicate that the decellularization protocol preserved the mechanical integrity required for vascular implantation.

### 4.3 *In vitro* cytocompatibility of DVGs

**FIGURE 3:**
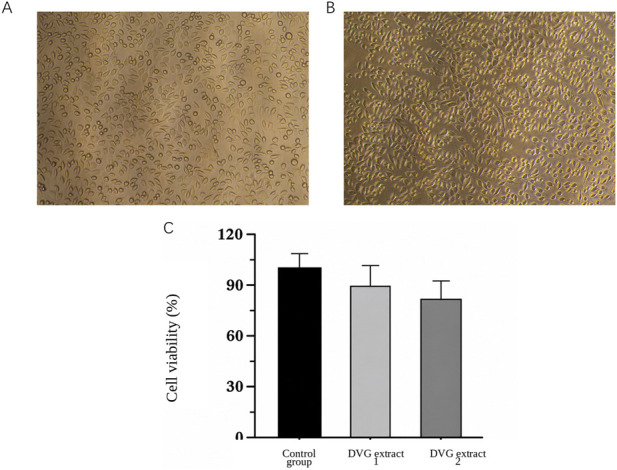
*In vitro* cytocompatibility of decellularized vascular graft extracts. **(A)** Representative cell morphology in the control group (culture medium only). **(B)** Representative cell morphology after exposure to decellularized graft extracts. **(C)** Quantification of cell viability (%). Data are presented as mean ± SD (n = 5 independent replicates per group). No statistically significant difference in cell viability was observed between the control group and the decellularized graft extract group (unpaired two-tailed Student’s t-test, p > 0.05). Error bars represent standard deviation (SD).

The cytocompatibility of DVGs was assessed using standard *in vitro* cytotoxicity assays in accordance with ISO guidelines. Cells exposed to extracts from decellularized grafts exhibited high viability comparable to that of the control group (Figures 3A,B). Quantitative analysis confirmed the absence of significant cytotoxic effects, indicating favorable biocompatibility of the decellularized porcine arteries (Figure 3C). These findings support the suitability of the grafts for subsequent *in vivo* evaluation.

### 4.4 *In vivo* patency following allogeneic implantation

**FIGURE 4:**
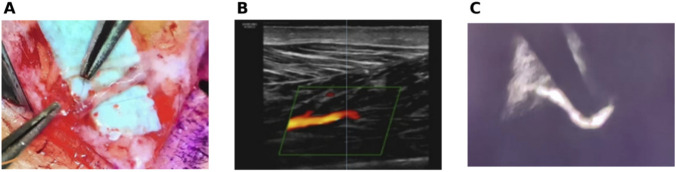
*In vivo* implantation and imaging confirmation of graft patency. **(A)** Representative intraoperative view during graft implantation/anastomosis. **(B)** Color Doppler ultrasound demonstrating flow signal within the implanted graft/target vessel. **(C)** Representative angiographic image confirming graft/vessel patency.

To evaluate *in vivo* performance, decellularized porcine carotid arteries were implanted as allogeneic vascular grafts. All surgical procedures were completed successfully without acute complications. During the 3-month implantation period, no evidence of graft rupture, thrombosis, or occlusion was observed. Explanted grafts maintained structural integrity and luminal patency, as confirmed by intraoperative observation, color Doppler ultrasound, and angiographic imaging (Figures 4A-C), indicating stable *in vivo* performance.

### 4.5 *In vivo* implantation and and qualitative morphological assessment

Following implantation, the DVGs remained patent during the 3-month observation period without obvious aneurysmal dilation, rupture, or gross thrombosis.

However, the present *in vivo* evaluation was based exclusively on morphological methods including histology and scanning electron microscopy, without immunohistochemical confirmation using markers such as CD31 or von Willebrand factor, and without functional assays. The following observations are therefore qualitative morphological evidence and do not confirm endothelialization or functional vascular integration.

**FIGURE 5:**
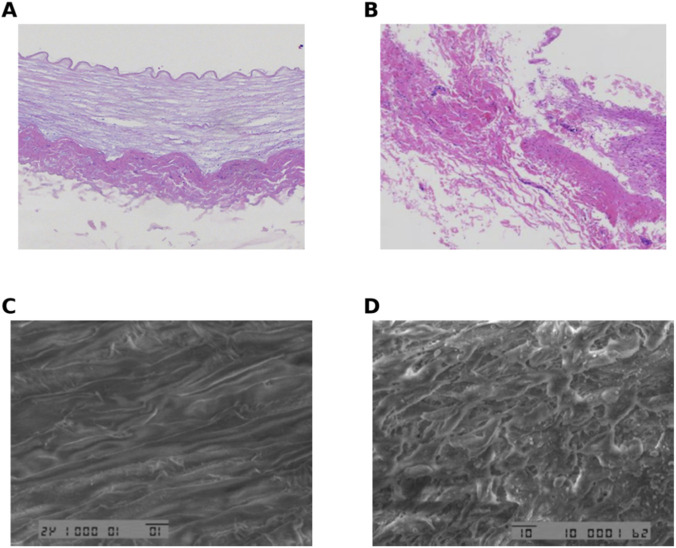
Histological remodeling and surface ultrastructure evaluation of explanted grafts. **(A,B)** Representative H&E-stained sections showing graft wall remodeling/cellular infiltration after implantation. **(C,D)** Scanning electron microscopy (SEM) images showing luminal surface morphology/coverage at different magnifications.

Histological examination demonstrated cellular coverage on the luminal surface and cellular infiltration within the graft wall, indicating host cell colonization. Quantitative assessment of luminal cell coverage, intimal thickness, and infiltrating cell density was not performed in this study. Newly populated cells exhibited organized distribution within the graft wall (Figures 5A,B). Scanning electron microscopy further demonstrated continuous cellular coverage on the luminal surface of the grafts (Figures 5C,D). Cells with endothelial-like morphology (flattened, polygonal shape) were observed on the luminal surface, along with spindle-shaped cells within the wall consistent with smooth muscle cell-like morphology. These morphological observations suggest that decellularized porcine arteries support host cell colonization and structural integration following implantation (Lin et al., 2018).

### 4.6 Hemocompatibility of DVGs

Hemolysis assay: The hemolysis ratio of the DVGs was markedly lower than that of the positive control and comparable to the negative control (Supplementary Figure S1). The calculated hemolysis value was below 5%, satisfying the ISO 10993-4 criterion for blood-contacting biomaterials, indicating negligible erythrocyte damage.

Coagulation assays: Coagulation parameters were measured before and after exposure to the decellularized grafts, with five independent replicates per time point (n = 5). As summarized in Table 1, all preoperative values were within normal ranges. Postoperative values remained comparable to preoperative levels for aPTT, PT, and TT (p > 0.05 for all, paired two-tailed Student’s t-test). The postoperative FIB level (0.97 ± 0.11 g/L) showed a slight but statistically significant decrease compared to preoperative (1.82 ± 0.15 g/L, p < 0.01), although the value was only marginally below the lower limit of the normal range (1.00–3.20 g/L) and was not associated with any clinical signs of abnormal bleeding or thrombotic risk. These results demonstrate that the decellularized scaffold does not significantly perturb the intrinsic or extrinsic coagulation pathways.

**TABLE 1:** Coagulation profiles before and after exposure to DVGs.

Parameter	Preoperative (n = 5)	Postoperative (n = 5)	Normal range
aPTT (s)	25.9 ± 2.1	29.7 ± 3.4	21.0–59.0
PT (s)	18.2 ± 1.5	20.7 ± 2.0	15.0–34.0
FIB (g/L)	1.82 ± 0.15	0.97 ± 0.12*	1.00–3.20
TT (s)	16.5 ± 1.8	28.5 ± 4.2	12.0–33.0

Data are presented as mean ± SD. *p < 0.05 vs. preoperative (paired t-test).

Platelet adhesion observation: Scanning electron microscopy was used to qualitatively assess platelet adhesion on the luminal surface of the DVGs. As shown in Figures 5C,D, only a limited number of platelets adhered to the graft surface. The majority of adherent platelets exhibited a rounded or discoid morphology without pseudopodia formation, spreading, or aggregation, indicating a non-activated state. This qualitative observation suggests that the decellularized ECM surface does not readily promote platelet adhesion or activation.

## 5 Discussion

### 5.1 Significance of effective decellularization and ECM preservation

Achieving efficient cell removal while preserving extracellular matrix architecture remains a key challenge in the development of DVGs. In this study, histological and immunohistochemical analyses confirmed the effective removal of endothelial and smooth muscle cells, with well-maintained collagen and elastin frameworks in porcine carotid arteries. Quantitative residual DNA measurement further supported these findings: decellularized grafts contained only 32.6 ± 5.8 ng/mg dry tissue DNA, well below the accepted 50 ng/mg threshold for reduced immunogenicity. Preservation of native ECM is critical, as it provides both mechanical support and bioactive cues essential for subsequent cell repopulation. Compared with synthetic grafts, ECM-based scaffolds retain tissue-specific biochemical signals that regulate cell adhesion, migration, and phenotypic stability. Collectively, our results indicate that the applied decellularization protocol achieves an appropriate balance between efficacy and structural preservation under the conditions tested.

### 5.2 Mechanical compliance and implications for vascular patency

Mechanical mismatch between vascular grafts and native vessels has been widely recognized as a major contributor to intimal hyperplasia and long-term graft failure. Excessive stiffness or reduced compliance can disturb local hemodynamics, leading to endothelial dysfunction and adverse remodeling. In this study, decellularized porcine arteries exhibited tensile behavior and burst pressure resistance comparable to those of native vessels under static and quasi-static testing conditions. These findings suggest that the intrinsic mechanical properties of the arterial ECM were largely preserved following decellularization. Importantly, maintenance of mechanical compliance may contribute to the favorable *in vivo* patency observed in the allogeneic implantation model, although dynamic compliance and fatigue resistance were not assessed. The ability of decellularized grafts to more closely mimic native arterial mechanics under the tested conditions represents a potential advantage over conventional synthetic materials, but further mechanical characterization is needed to establish functional equivalence.

### 5.3 Hemocompatibility of DVGs

Hemocompatibility is essential for small-diameter vascular grafts. Here, the decellularized porcine carotid arteries exhibited a hemolysis ratio below 5%, meeting ISO 10993-4 requirements. Consistent with a recent report (Mahara et al., 2024), the observed minimal hemolysis confirms the absence of erythrocyte-damaging residues. Coagulation parameters (PT, aPTT, FIB, TT) remained within normal ranges, indicating no activation of intrinsic or extrinsic coagulation pathways, aligning with studies showing that decellularized porcine matrices preserve native coagulation profiles when residual detergents are adequately removed (Elbaz and Sholzberg, 2020). Qualitative SEM observation revealed limited platelet adhesion with non-activated morphology, contrasting with reports that decellularized matrices can act as platelet-activating surfaces (Kasimir et al., 2017); the favorable response in our study likely reflects preserved native ECM architecture and effective post-decellularization washing. Unlike synthetic grafts (e.g., ePTFE, PET), which often require heparin or endothelial coatings to reduce thrombogenicity (Ham et al., 2021), decellularized grafts may offer inherent low thrombogenicity based on these *in vitro* observations.

### 5.4 Morphological evidence of luminal cell coverage

The native endothelium plays critical roles in regulating thrombosis, inflammation, and smooth muscle behavior (Liu and Fan, 2025). However, the present study evaluated luminal cell coverage exclusively through morphological methods (histology and SEM) without immunohistochemical or functional validation. Therefore, the observations described below should be interpreted as preliminary morphological evidence of host cell colonization, not as confirmed functional endothelialization or vascular integration.

The present study demonstrated morphological evidence of luminal cellular coverage and cell infiltration into the graft wall following 3 months of implantation. The morphology of luminal cells was consistent with that of endothelial-like cells (i.e., flattened, polygonal shape), and the presence of spindle-shaped cells within the wall was consistent with smooth muscle cell-like morphology. We emphasize that these are qualitative morphological observations only; we did not perform immunohistochemical staining for endothelial markers (e.g., CD31, von Willebrand factor), smooth muscle markers (e.g., α-SMA, calponin), or functional assays (e.g., barrier function, nitric oxide production, or anti-thrombotic activity). Therefore, while the decellularized porcine ECM appears to support host cell colonization, statements regarding ‘endothelialization’ or ‘vascular integration’ should be interpreted as preliminary morphological findings rather than confirmed functional outcomes. Unlike pre-seeded tissue-engineered grafts, decellularized grafts rely on host-mediated recellularization, which may offer practical advantages if functional integration can be demonstrated in future studies. These morphological observations suggest that decellularized porcine arteries support host cell colonization and structural changes consistent with integration, but do not demonstrate functional vascular regeneration.

### 5.5 Immunological considerations and graft integration

Immune responses to implanted biomaterials can profoundly influence graft remodeling and long-term performance. Although decellularization significantly reduces immunogenicity by removing cellular antigens, residual ECM components may still modulate host immune responses (Wu SL. et al., 2025). In this study, no signs of acute graft failure or severe adverse reactions were observed during the implantation period. Histological findings suggested progressive tissue integration rather than fibrotic encapsulation. These results are consistent with previous reports indicating that decellularized ECM can promote a constructive remodeling response rather than chronic inflammation. Nevertheless, further investigations focusing on immune cell phenotypes and long-term inflammatory profiles are warranted to fully characterize host–graft interactions.

### 5.6 Mechanistic insights into ECM-mediated regeneration and host response

Beyond structural preservation, DVGs retain ECM signals that actively orchestrate host cell behavior (Badylak et al., 2009). Preserved ECM components such as collagen IV, laminin, and glycosaminoglycans provide integrin-binding motifs like RGD sequences that engage endothelial and smooth muscle cell surface integrins (Davis and Senger, 2005). Upon ligation, these integrins trigger intracellular signaling cascades, particularly the FAK/PI3K/Akt and MAPK/ERK pathways, which regulate cell adhesion, migration, proliferation, and survival (Sieg et al., 2000). Recent studies have also linked YAP/TAZ mechanotransduction via β1-integrin to vascular cell remodeling and endothelial stabilization (Totaro et al., 2018). Thus, the preserved collagen and elastic fibers observed in our grafts could potentially serve as both a scaffold and a signaling platform, although this remains speculative without direct molecular evidence.

In addition, the decellularization process may expose cryptic bioactive epitopes called matricryptins that are normally hidden within native ECM proteins (Ricard-Blum and Vallet, 2016). These cryptic sites can promote angiogenesis and endothelial cell migration (Davis et al., 2002). Furthermore, matrix-bound growth factors such as VEGF and bFGF that survive decellularization may be released upon cell-mediated ECM remodeling, providing local regenerative cues (Hynes, 2009).

Host immune modulation is equally critical. Macrophages undergo a phenotypic transition from a pro-inflammatory M1 state to a pro-regenerative M2 state following implantation of decellularized scaffolds (Brown and Badylak, 2014). M2 macrophages secrete anti-inflammatory cytokines like IL-10 and TGF-β as well as matrix metalloproteinases that facilitate ECM degradation and host cell infiltration, ultimately promoting tissue integration (Sadtler et al., 2016). The low residual DNA content in our grafts, which fell below 50 ng/mg, may further reduce activation of pro-inflammatory Toll-like receptor pathways and favor an M2-skewed response (Keane et al., 2012). Although direct immunophenotyping was not performed in this study, the absence of chronic inflammation and the progressive tissue integration we observed are consistent with such a constructive remodeling environment.

### 5.7 Limitations of the present study

Several limitations should be acknowledged. First, the 3-month implantation period captures only early graft remodeling; longer-term studies are needed to assess durability, late-stage vascular adaptation, and possible calcification. Second, the allogeneic porcine model does not fully replicate the human immune environment. Third, quantitative assessments—including endothelial coverage (CD31), intimal hyperplasia thickness, and cell infiltration density—were not performed. Fourth, a comprehensive immunological characterization is lacking: we did not quantify residual DNA, assess xenogeneic epitopes such as α-Gal, or evaluate inflammatory cell infiltration (CD68^+^ macrophages and CD3^+^ T-cells). Fifth, quantitative platelet adhesion analysis was not performed; only qualitative observation was conducted. Sixth, and importantly, this study does not provide evidence of functional vascular regeneration (e.g., endothelium-dependent vasodilation, anti-thrombotic activity, or barrier function). All *in vivo* observations of cellular coverage and infiltration are morphological in nature and should be distinguished from true functional integration. Consequently, our *in vivo* observations support preliminary biocompatibility rather than definitive efficacy or immune safety. Any references to translational potential in this manuscript should be interpreted as hypothesis-generating observations that require validation in future studies with appropriate controls and quantitative endpoints. Despite these limitations, the present work provides an integrative characterization of decellularized porcine carotid arteries, including histological validation, mechanical testing, cytocompatibility, hemocompatibility, and short-term *in vivo* observation.

### 5.8 Future directions and remaining challenges

DVGs represent an active area of investigation bridging synthetic prostheses and tissue-engineered constructs. The present findings provide preliminary evidence supporting the feasibility of producing porcine decellularized carotid arteries with preserved ECM architecture, baseline mechanical integrity, and hemocompatibility. However, we emphasize that these observations are based on a limited *in vivo* study without control groups or quantitative remodeling assessment. Substantial additional preclinical validation, including long-term implantation studies, comparative evaluations against existing clinical standards (e.g., autologous vein or synthetic grafts), and quantitative analyses of remodeling outcomes, will be required before any translational claims can be substantiated.

Porcine arteries offer a practical and clinically relevant source: their anatomy and mechanics closely resemble human vessels, they are readily available, and can be processed under standardized conditions. Unlike cell-seeded grafts, acellular DVGs reduce manufacturing complexity and cost while relying on host-mediated recellularization for functional integration (Khan et al., 2017).

Nevertheless, several challenges remain. Long-term implantation studies are needed to assess durability, calcification resistance, and late immune responses. Optimization of decellularization protocols to standardize ECM composition and minimize residual immunogenic components is essential. Advanced characterization (proteomic profiling, biomechanical fatigue testing) may provide deeper structure-function insights. Future research should explore functional endothelial assessment (antithrombotic activity, vasoregulation) and host-graft immune interactions. Targeted surface modifications (e.g., heparinization (Li et al., 2024)) and bioactive cues (mesenchymal stem cells, bFGF, VEGF (Ahmed et al., 2021; Bertanha et al., 2014)) offer promising avenues for enhancing graft performance.

In summary, this study supports the feasibility of porcine DVGs as candidate scaffolds for further preclinical investigation, but does not establish translational readiness. Continued optimization and rigorous validation are necessary before clinical application can be considered.

## 6 Conclusion

This study demonstrates the feasibility of producing decellularized porcine carotid arteries with preserved ECM architecture, baseline mechanical integrity, and hemocompatibility using a detergent-enzymatic protocol. Residual DNA content fell below the 50 ng/mg threshold, and *in vitro* cytocompatibility and hemocompatibility were favorable. Preliminary *in vivo* observations over 3 months indicated graft patency and morphological evidence of host cell colonization, reflecting structural changes rather than confirmed functional vascular regeneration. However, given the study’s limitations, including the absence of control groups, small sample size, lack of quantitative morphological assessment, and absence of functional endothelial validation, these findings should be considered hypothesis-generating rather than conclusive. Throughout this manuscript, the term ‘remodeling’ refers to structural changes observed histologically, not to verified functional vascular regeneration. Further studies with extended implantation durations, control groups, quantitative outcome measures, and functional assays (e.g., endothelial marker staining, anti-thrombotic activity) are required to determine whether this approach has value for vascular reconstruction.

## Data Availability

The raw data supporting the conclusions of this article will be made available by the authors, without undue reservation.
