# Supplementary material for: Decellularized porcine vascular grafts: structural integrity and translational potential for allogeneic implantation
Source: Front Bioeng Biotechnol. 2026 May 26;14:1823315. doi: 10.3389/fbioe.2026.1823315 (PMC13246665; doi:10.3389/fbioe.2026.1823315)

**Supplementary figure 1.** Hemolysis assay of decellularized vascular grafts (DVGs). (A) Representative visual appearance of the positive control (deionized water), the experimental group (DVG extract), and the negative control (PBS). (B) Quantitative analysis of hemolysis percentage. Data are presented as mean ± SD (n = 5 per group). The positive control exhibited 100% hemolysis (by definition), the negative control showed negligible hemolysis (1.0 ± 0.2%), and the DVG group displayed a hemolysis ratio of 2.1 ± 0.3%, which is well below the ISO 10993‑4 acceptable limit of 5% for blood‑contacting biomaterials. ***p < 0.001 compared to the positive control (one‑way ANOVA with Tukey’s post‑hoc test); n.s. indicates no significant difference between the DVG group and the negative control (p > 0.05). Error bars represent standard deviation (SD).


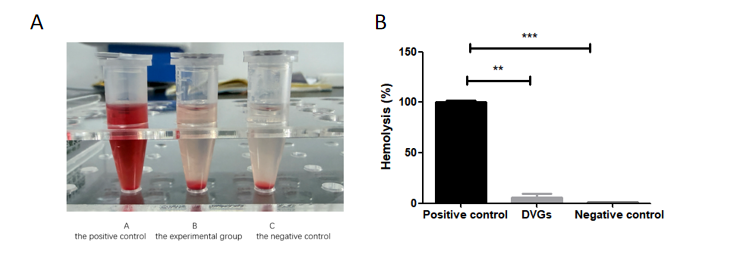

Supplement: Supplementary file 1 [file Supplementaryfile1.docx]
